# Dominant-Negative Effects of Adult-Onset Huntingtin Mutations Alter the Division of Human Embryonic Stem Cells-Derived Neural Cells

**DOI:** 10.1371/journal.pone.0148680

**Published:** 2016-02-10

**Authors:** Carla Lopes, Sophie Aubert, Fany Bourgois-Rocha, Monia Barnat, Ana Cristina Rego, Nicole Déglon, Anselme L. Perrier, Sandrine Humbert

**Affiliations:** 1 Grenoble Institut des Neurosciences, Grenoble, France; 2 INSERM U836, Grenoble, France; 3 Grenoble Alpes University, Grenoble, France; 4 CNC-Center for Neuroscience and Cell Biology, University of Coimbra, Coimbra, Portugal; 5 Institute for Interdisciplinary Research of the University of Coimbra (IIIUC), Coimbra, Portugal; 6 CECS, I-STEM, AFM, Corbeil-Essonnes, France; 7 Inserm U861, I-STEM, AFM, Corbeil-Essonnes, France; 8 UEVE U861, I-STEM, AFM, Evry, France; 9 Faculty of Medicine, University of Coimbra, Coimbra, Portugal; 10 Lausanne University Hospital (CHUV), Department of Clinical Neurosciences (DNC), Laboratory of Cellular and Molecular Neurotherapies (LNCM), Lausanne, Switzerland; Inserm U837, FRANCE

## Abstract

Mutations of the huntingtin protein (HTT) gene underlie both adult-onset and juvenile forms of Huntington’s disease (HD). HTT modulates mitotic spindle orientation and cell fate in mouse cortical progenitors from the ventricular zone. Using human embryonic stem cells (hESC) characterized as carrying mutations associated with adult-onset disease during pre-implantation genetic diagnosis, we investigated the influence of human HTT and of an adult-onset HD mutation on mitotic spindle orientation in human neural stem cells (NSCs) derived from hESCs. The RNAi-mediated silencing of both *HTT* alleles in neural stem cells derived from hESCs disrupted spindle orientation and led to the mislocalization of dynein, the p150^*Glued*^ subunit of dynactin and the large nuclear mitotic apparatus (NuMA) protein. We also investigated the effect of the adult-onset HD mutation on the role of HTT during spindle orientation in NSCs derived from HD-hESCs. By combining SNP-targeting allele-specific silencing and gain-of-function approaches, we showed that a 46-glutamine expansion in human HTT was sufficient for a dominant-negative effect on spindle orientation and changes in the distribution within the spindle pole and the cell cortex of dynein, p150^*Glued*^ and NuMA in neural cells. Thus, neural derivatives of disease-specific human pluripotent stem cells constitute a relevant biological resource for exploring the impact of adult-onset HD mutations of the *HTT* gene on the division of neural progenitors, with potential applications in HD drug discovery targeting HTT-dynein-p150^*Glued*^ complex interactions.

## Introduction

Huntington’s disease (HD) is an autosomal dominant neurodegenerative disorder caused by abnormal extension of a tract of CAG repeats in the first exon of the *HTT* gene [1]. Mutated forms of the huntingtin (HTT) protein carry an extended stretch of glutamine residues (polyQ) close to the N-terminus [2]. The mean size of the CAG expansion is 18 repeats in the general population, but patients with HD carry expansions including more than 35 CAG repeats. Most forms of HD patients have an onset during adulthood. For such forms, the longest CAG expansion of the two *HTT* alleles contains 41 to 48 repeats, with a mean of 44 CAG repeats [3], [4], [5]. The affected patients are clinically characterized by psychiatric, cognitive and motor disturbances beginning between the ages of 35 and 50 years. Early onset of the symptoms, high severity and rapid disease progression are associated with the presence of larger numbers of CAG repeats [6]. Fewer than 10% of patients develop symptoms before the age of 20 years; this juvenile form of the disease is characterized by a more widespread and rapidly progressing pattern of brain degeneration associated with a larger number (> 60) of CAG repeats than for adult-onset HD [7].

HTT is a large scaffold protein involved in diverse cellular functions in multiple cellular compartments [8]. HTT interacts with hundreds of protein partners. It interacts directly with dynein and indirectly with dynactin, through huntingtin-associated protein 1 (HAP-1), which binds to p150^*Glued*^. This complex regulates the microtubule-dependent transport of organelles in neurons [9], [10] and ciliogenesis [11], and ensures the correct orientation of the mitotic spindle, thereby regulating cell fate [12], [13]. Current knowledge of the pathological processes involved suggests that neuron loss in HD results from both a loss of wild-type HTT (wtHTT) functions and the gain of new toxic functions by the mutant HTT protein (mutHTT), probably acting in both cell-autonomous and non-autonomous manners [14].

Most of the genetic mouse and cellular models used to study the impact of HTT mutations express mutHTT fragments or full-length mutHTT with particularly long polyQ stretches encoded by expansions of more than 100 CAG repeats. The extent to which “acute” models of this kind can accurately replicate the decades-long pathological processes resulting from the most common types of HD mutations (41–48 CAGs) observed in the brains of patients is difficult to ascertain. Technologies providing access to human pluripotent stem cells (hPSCs) [15] have made it possible to develop new disease models based on hPSC derivatives that could potentially bridge this gap and improve our understanding of HD pathogenesis. A number of disease-specific (i.e. mutant) hPSC lines have been obtained from embryos found to carry mutations during a pre-implantation genetic diagnosis procedure or from somatic cells from patients. Several of these lines carry CAG repeats of lengths known to be associated with the adult-onset form of the disease and corresponding to the most common form of HD mutation. Such HD-hPSC lines have been used to decipher the impact of adult- or juvenile-onset HD mutations of the *HTT* gene in human cells [16], [17], [18], [19], [20], [21].

HTT regulates the division of mouse embryonic cortical progenitors and mammary stem cells [13], [12]. The consequences of HTT mutation for cell division of neuronal progenitors have recently been deciphered in the context of embryonic cortical development, in a mouse genetic model carrying an *HTT* mutation with an expansion of more than 100 CAG repeats [22]. In this study, we combined the use of neural derivatives of wild-type (WT) and adult-onset HD-hESCs and SNP-targeting *HTT* allele-specific mRNA interference to investigate the role of human HTT in the division of neural progenitors and to determine whether an adult-onset HD mutation affects this function.

## Materials and Methods

### Cell culture

Neural cells were derived from H9 (WT XX, passages 40–60, WiCell Research Institute) [15], SIVF018 (XX, 46 CAG, passage 18–30, Sydney IVF Stem Cells, Australia) [23] and SA01 (WT XY, passages 12, CellArtis AB, Göteborg, Sweden) [24] embryonic stem cell lines, as previously described in [25]. Neural stem cells (NSC) obtained from hESCs were maintained on poly-L-ornithine and laminin (Sigma, St. Louis, Missouri, USA) coated plates until passage 29 and then discarded. Cells were harvested with 0.05% trypsin-EDTA (Invitrogen, Cergy Pontoise, France) and seeded at 100x10^3^ cells/cm^2^ in culture plates. NSC were cultured in 1:1 ratio of Neurobasal: DMEM/Hams’s F-12 (Invitrogen, Cergy Pontoise, France), supplemented with 0.1% penicillin/streptomycin, 0.1% β-mercaptoethanol (Sigma-Aldrich), 1% B27 (Invitrogen, Cergy Pontoise, France), 0.5% N_2_ (Invitrogen, Cergy Pontoise, France), supplemented with 10 ng/mL of basic fibroblast growth factor (FGF-2) (Invitrogen, Cergy Pontoise, France) and 10 ng/mL epidermal growth factor (EGF) (R&D systems, Minneapolis, USA). Cells were plated in multiwell plates 54 hr before synchronization using RO-3306 (10 μM, for 18 hr) (Enzo Life Sciences, France). The population was released from the G2 block by three washes with pre-warmed drug-free media and incubation with fresh media (30 min).

### Plasmids and siRNA

Genotypes on exon 50 for SNP rs362331 of H9 and SIVF018 NSC lines were analysed by sequencing the PCR product encompassing this SNP generated using the following primers: 5’-CCCCAAACGAAGGTACACGA-3’ and 5‘- CCTGTTGGCCATCTCTCACC-3’. SIVF018 NSC line is heterozygous at SNP rs362331 (C/T), while H9 is homozygous (T/T) (S1A Fig). shRNAs targeting these SNP (si50C, si50T) were designed as previously described [16]. The SIN-CWP-GFP-LTR(N)-TRE-si1.1 (complete knock-down, sh*HTT1*.*1*.), SIN-CWP-GFP-LTR(N)-TRE-si50T (50T allele-specific, sh*HTT50T*), SIN-CWP-GFP-LTR(N)-TRE-si50C (50C allele-specific, sh*HTT50C*) and SIN-CWP-GFP-LTR(N)-TRE-siUNIV (shcontrol) plasmids were previously described [16]. The plasmids encoding N-terminal fragments of wild-type (23Q; mCherry-HTT-N586-23Q) and polyQ HTT (100Q; mCherry-HTT-N586-100Q) constructs were previously described [26].

### Transfection

Prior to nucleofection, cells were harvested with trypsin; 5x10^6^ cells were nucleofected using the Amaxa Rat NSC Nucleofector Kit (Lonza) according to the manufacturer protocol. NSC were resuspended in 100 μL of nucleofection solution with 5 μg of DNA and 0.5 μg of pmaxGFP Vector or 1.5 μg (1 μM) of siRNA and electroporated with the Amaxa Nucleofector Program A-033 (Lonza) before plating on poly-ornithine/laminin coated dishes. For shRNA nucleofection, five million NSC resuspended in 100 μL of nucleofection solution of the Amaxa^®^ Rat NSC Nucleofector^®^ Kit (Lonza) with 5 μg of shRNA (1 μM) were electroporated with the Amaxa Nucleofector Program A-033 (Lonza) before plating on poly-ornithine/laminin coated dishes. All cells were incubated in humidified 37°C/5% CO_2_ incubator and medium was changed after 24 hr.

### RNA extraction and Quantitative RT-PCR

Total RNA from NSC cells was extracted using RNeasy Mini Spin Columns (Qiagen, Courtaboeuf, France). The purified RNA was quantified with NanoDrop ND-1000A spectrophotometer. Transcription was performed on 500 ng of RNA using Cloned AMV First-Strand cDNA Synthesis Kit (Invitrogen, Cergy Pontoise, France). cDNA synthesis was performed using total RNA primed with oligo(dT) (50 μM) mixed with Random Hexamers (50 ng/μL) according to manufacturer’s protocol. Quantitative real time-polymerase chain reactions (QRT-PCRs) are performed with Power SYBR Green PCR Mix and a LC480 system (Roche, Boulogne-Billancourt, France). Quantification was performed at a threshold detection line (Ct value). The Ct of each target gene was normalized to the 18S housekeeping gene. For primer sequences see S1 Table.

*HTT* mRNA allelic ratio analysis was performed using TaqMan SNP Genotyping Assays (Hs00918153_m1: Applied Biosystems, Foster City, CA, USA) on cDNA used for QRT-PCR. The median of difference of Ct of each target allele (3 technological replicate) was used to calculate the *HTT* allelic ratio expressed in % of SNP50C and SNP50T in all *HTT* mRNA (genomic DNA was used as a control sample with 50%/50% *HTT* allelic ratio).

For the CAG repeats size analysis, a PCR was performed using TaKaRa LA Taq DNA Polymerase with GC Buffers (Takara Bio, Madison, USA), according to manufacturer protocol, on cDNAs obtained from allele-specific cDNA synthesis using specific primers for *HTT* exon 1 5′-AAGGCCTTCGAGTCCCTCAA-3’ and 5’-CACACGGTCTTTCTTGGTAGC-3’. The expected amplicon size for a normal allele with 23 CAG repeats is 281bp. The PCR products were analysed by the Bioanalyser 2100 (Agilent, CA, USA) using microfluidic chips which were prepared according to manufacturer’s instructions. Analysis of the data was performed using Data Review software version A.01.20 Sl211 and presented in the form of electropherograms or alternatively as a list of calculated band sizes.

### Immunofluorescence

To visualize spindles, cells were permeabilized with PHEM/0.5% Triton X-100 for 30 sec, then fixed with 4% PAF/PHEM (20 min at RT) and with methanol at -20°C (5 min). Cells were rehydrated with PBS/0.1% Triton X-100 (3 washes), blocked in 10% Normal Goat Serum (NGS)/PBS (30 min) and incubated with the following antibodies: anti-γ-tubulin (1:500, T3320 or 1:100, 6557; Sigma-Aldrich); anti-p150^*Glued*^ (1:100, 610474; BD Transduction Laboratories); anti-736-HTT (1:300; [27]); anti-NuMA (1:500; Novus biological).

For dynein intermediate chain (DIC) staining (1:200; mAB 1618 Chemicon) the fixation procedure was as follows: cells were immersed in microtubule stabilizing buffer (MTSB: 4 M glycerol, 100 mM PIPES, pH 6.8, 1 mM EGTA, 5 mM MgCl_2_) for 2 min, followed by extraction for 2 min in MTSB/0.5% Triton X-100. Cells were then fixed for 3 min with methanol at -20°C.

For all immunostainings, the slides were counterstained with DAPI (Roche, Boulogne-Billancourt, France) and mounted in Mowiol. The images were captured either with a three-dimensional deconvolution imaging system or with a Leica DM RXA microscope equipped with a 63x oil-immersion objective coupled to a piezzo and a Micromax RTE/CDD-1300-Y/HS camera controlled by Methamorph software (Molecular Devices). Z-stack steps were of 0.3 μm. Images were treated with ImageJ (http://rsb.info.nih.gov/ij/, NIH, USA).

### Spindle Orientation Quantification and Image Analyses

Spindle orientation in metaphase cells stained for γ-tubulin and DAPI to visualize the spindle poles and chromatin was quantified using ImageJ software (http://rsb.info.nih.gov/ij/, NIH, USA). A line crossing both spindle poles was drawn on the Z projection pictures and repositioned along the Z-axis using the stack of Z-sections. The angle between the pole-pole and the substratum plane was calculated by the ImageJ Plug-in. Spindle lengths and cell lengths were quantified along the line that crosses both spindle poles. In rescue experiments, cells that were positive for mCherry (thus expressing N-terminal fragments of wild-type and mutant HTT) were analysed for spindle orientation.

The quantification of p150^*Glued*^ and DIC at spindle poles was achieved with 3D object counter plugin ([28]; http://imagej.nih.gov/ij/plugins/track/objects.html). A circle (radius equal to half of pole-pole distance) was drawn around the pole position, and image was cleared outside the circle. Total volume and intensity of the particles were retrieved for further analysis.

Quantification of NuMA at spindle pole was performed on the resulting z-stacks (one for each pole) using JACoP plugin (http://imagej.nih.gov/ij/plugins/track/jacop.html) which determines the localization of NuMA at spindle poles given by γ-tubulin. Manders' overlap coefficient corresponds to the co-localization between both images.

### Immunoblotting

Cells were lysed 72 h after transfection (lysis buffer: 50 mM Tris-HCl pH 7.5, 0.1% Triton X-100, 2 mM EDTA, 2 mM EGTA, 50 mM NaF, 10 mM β-glycerophosphate, 5 mM sodium pyrophosphate, 1 mM sodium orthovanadate, 0.1% (v/v) β-mercaptoethanol, 250 μM PMSF, and 10 mg/ml aprotinin and leupeptin). Twenty five μg of proteins were loaded in 6% gel, subjected to SDS-PAGE and electrophoretically transferred to Protran nitrocellulose membranes from Whatman. Blots were blocked in 5% BSA/TBST buffer (20 mM TrisHCl, 0.15 M NaCl, 0.1% Tween 20) and incubated with anti-dynein intermediate chain (DIC, 1:500; Millipore Bioscience Research Reagents), anti-HTT D7F7 (1:1000; Cell Signaling Technology), anti-polyQ 1C2 (1:5000; Euromedex), anti-NuMA (1:1000; Novus biological), anti-p150^*Glued*^ (1:1000; BD Transduction laboratories) and anti-α-tubulin (1:1000; Sigma) antibodies, for 1 hr.

Primary polyclonal antibodies used were: anti-PAR3 (1:1000), anti-PAR6 (1:500), anti-aPKC (1:1000), anti-RAB11A (1:500) and anti-mCherry (1:1000; Institut Curie, Paris).

### Statistical Analyses

GraphPad Prism 5.0 software (San Diego, CA, USA) was used for statistical analysis. Data are expressed as mean ± SEM of the indicated number of independent experiments. Statistical significance was analysed using parametric tests, namely Student’s t-test and one way-ANOVA.

## Results

### Human HTT regulates spindle orientation in dividing neural stem cells derived from human embryonic stem cells

**Fig 1:**
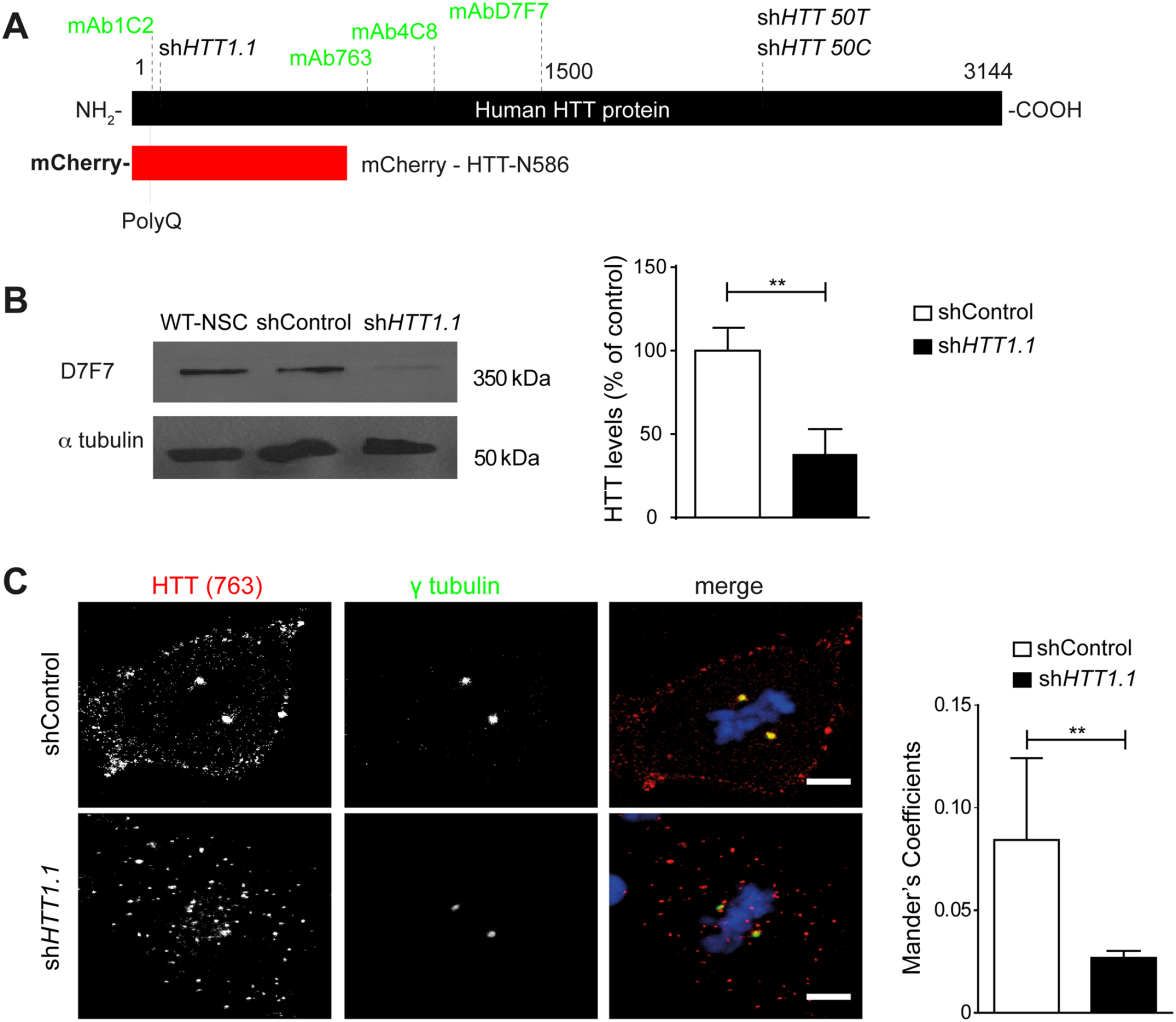
Silencing of HTT in wild-type human neural stem cells. (A) Schematic representation of HTT. (B) Western blotting with anti-HTT and anti-α-tubulin antibody of lysates of H9-derived NSC treated with shControl and sh*HTT1*.*1*. The graph represents the quantitative assessments of the ratio of HTT over tubulin in each condition (3 independent experiments). (C) Immunostaining of metaphase shControl and sh*HTT1*.*1*-treated cells with anti-γ-tubulin and anti-HTT antibodies and DAPI counterstaining. Nuclei are counterstained with DAPI. Scale bars, 5 μm. The graph represents the quantification of anti-HTT signal at the spindle poles (*n* = 20 cells analysed in 3 independent experiments). Results are shown as the mean values ± SEM. **p<0.01; Student’s t-test.

We used NSCs differentiated from control human embryonic stem cells (the H9 line) and plasmids encoding a short hairpin RNA (shRNA) directed against *HTT* (sh*HTT1*.*1*) or a control shRNA (shControl) to study the effect of HTT knockdown (Fig 1A) [19]. HTT protein levels on western blots were 70% ± 16.18 (*n* = 3) lower in H9-derived WT-NSC 72 hours after transfection with the sh*HTT1*.*1* plasmid (Fig 1B). During mitosis, the polyclonal antibody (pAb) against HTT (763) labeled HTT at the spindle poles, as previously described (Fig 1C). HTT was also detected in the cell cortex. Treatment with sh*HTT1*.*1* significantly decreased the signal intensity at the spindle pole and resulted in the dispersion of HTT from its cortical location. In sh*HTT1*.*1* conditions, remaining HTT appeared more disperse in the cytoplasm forming larger punctae of unknown nature.

**Fig 2:**
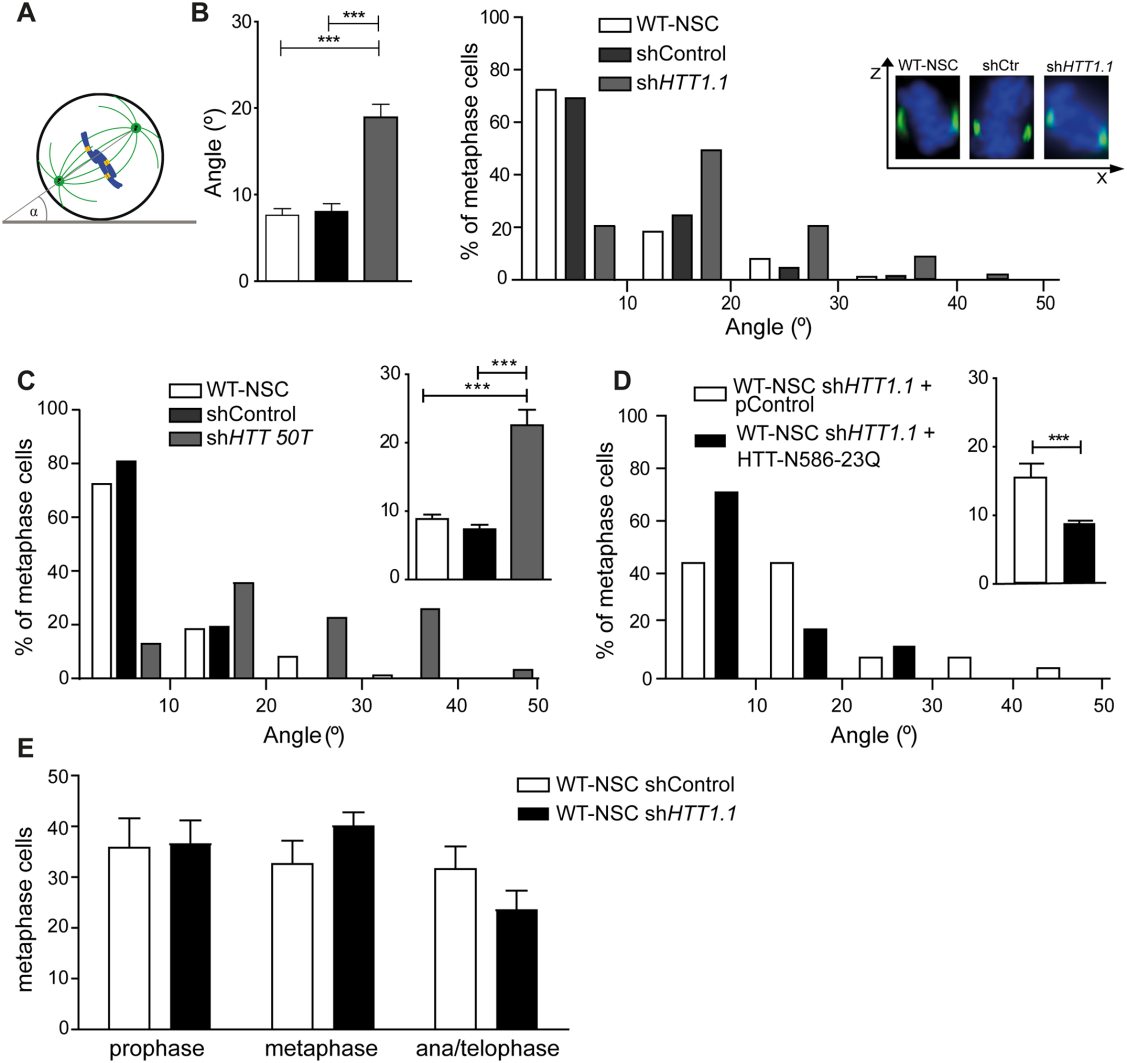
Silencing of human HTT induces spindle misorientation in human NSC. (A) Schematic illustration of spindle angle measurement. (B-D) H9-hESC derived neural stem cells were either non-treated or treated with shControl, sh*HTT1*.*1*, sh*HTT50T* or sh*HTT1*.*1*+N586-HTT-Q23 as indicated. Distribution and average of spindle angles of metaphase cells are shown. In B, cells were stained with anti-γ-tubulin and DAPI and Z-X projections are shown. (B) WT-NSC: *n* = 86, shControl: *n* = 65, sh*HTT1*.*1*: *n* = 74. (C) WT-NSC: *n* = 129, shControl: *n* = 108, sh*HTT50T*: *n* = 32. (D) WT-NSC sh*HTT*1.1 + pControl: *n* = 25, WT-NSC sh*HTT1*.*1* + HTT-N586-23Q: *n* = 49. (E) Distribution of H9-derived WT-NSC transfected with shControl and sh*HTT1*.*1* in prophase, metaphase and ana/telophase (WT-NSC shControl: *n* = 353, WT-NSC sh*HTT1*.*1*: *n* = 389). All data are from at least 3 independent experiments. Results are shown as the mean values ± SEM. ***p<0.001; **p<0.01; Student’s t-test or one-way ANOVA.

We then carried out immunostaining with an anti-γ-tubulin antibody, of H9-derived WT-NSCs transfected with shControl or sh*HTT1*.*1* plasmids, for analysis, in Z-series stacks (ΔZ > 1.8 μm), of the position of the spindle poles with respect to the substratum plane (Fig 2A and 2B). Spindle orientation was determined in metaphase cells, by measuring the angle between the pole-pole axis (the axis of the metaphase spindle) and the substratum plane. Non-transfected WT-NSCs and WT-NSCs transfected with shControl had a significantly smaller mean angle (7.6° ± 0.7, *n* = 86; 8.1° ± 0.9, *n* = 65, respectively) than sh*HTT1*.*1*-treated cells (18.9° ± 2.1, *n* = 74) (Fig 2B). The proportion of metaphase cells with an angle greater than 10° was higher in sh*HTT1*.*1*-transfected WT-NSCs (79.5%) than in control cells (non-transfected cells: 28%; shControl-treated cells: 30%). A large proportion of the cells treated with sh*HTT1*.*1* (30.1%) had spindle angles exceeding 20°.

These results were confirmed with an alternative RNA interference plasmid targeting the rs362331 SNP in exon 50 of the *HTT* gene in the H9 genome (sh*HTT50T*) (Fig 1A). The genotyping of H9 cells revealed that the H9 WT-hESC line was homozygous for the rs362331 SNP in exon 50 (T/T) (S1A Fig). We therefore transfected H9-derived WT-NSCs with sh*HTT50T* (S1B Fig), thereby targeting both alleles of *HTT* in H9-hESC derivatives. H9-derived WT-NSCs treated with shControl had mean spindle angles of less than 10° (8.9° ± 0.7, *n* = 129; 7.4° ± 0.6, *n* = 106), whereas the mean angle in sh*HTT50T*-treated WT-NSCs exceeded 20° (22.4° ± 2.3, *n* = 32) (Fig 2C). In sh*HTT50T*-treated cells, spindle angles were more evenly distributed, with more than 33% of the metaphase cells having an angle greater than 20°.

We then introduced a 586-amino acid N-terminal fragment of human HTT with 23 glutamine residues (N586-HTTQ23) into WT-NSCs, to reproduce HTT function during mitotic spindle orientation. The nucleotide sequence encoding this fragment was modified to ensure that it was not targeted by sh*HTT1*.*1*, which specifically targets endogenous human *HTT* [26]. N586-HTTQ23 rescued the spindle orientation defect observed after HTT silencing in WT-NSCs (Fig 2D).

We also analysed the distribution of cells across mitotic phases on the basis of chromosome configurations. HTT-depleted and control WT-NSCs were distributed in a similar manner across mitotic phases (Fig 2E). These findings indicate that human HTT regulates spindle orientation with no effect on progression through the cell cycle in human neural stem cells.

### HTT is required for the mitotic localization of dynein, dynactin and NuMA in human NSCs

**Fig 3:**
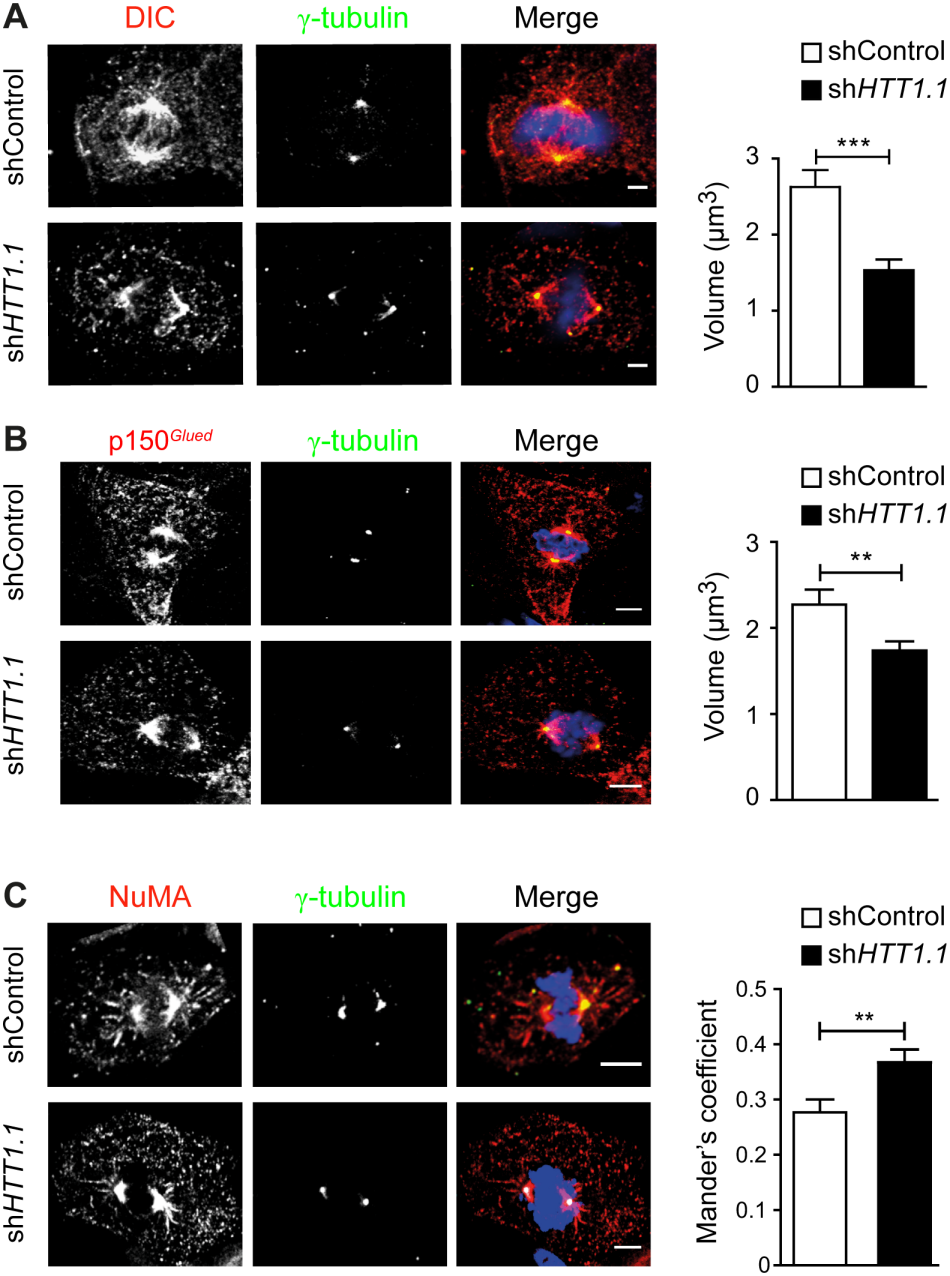
Loss of HTT alters dynein, p150^*Glued*^, and NuMA localization in human NSC. Immunostainings of metaphasic neural stem cells with anti-DIC (A), anti-p150^*Glued*^ (B), anti-NUMA (C) and anti-γ-tubulin antibodies. Nuclei are counterstained with DAPI. Scale bars, 5 μm. Graphs correspond to the quantifications of (A): shControl: *n* = 52 and sh*HTT1*.*1*: *n* = 60; (B): shControl: *n* = 57 and sh*HTT1*.*1*: *n* = 60; (C): shControl: *n* = 22 and sh*HTT1*.*1*: *n* = 22 cells analysed in at least 3 independent experiments. Results are shown as mean values ± SEM. ***p<0.001; **p<0.01; Student’s t-test.

HTT associates with dynein/dynactin to stimulate axonal transport in neurons [9]. The silencing of mouse HTT in neuronal cells and mammary stem cells leads to mislocalization of dynein, the p150^*Glued*^ subunit of dynactin and the large nuclear mitotic apparatus protein NuMA during mitosis, suggesting that HTT may act as a scaffold for these proteins, to ensure correct spindle orientation [12], [13]. We immunostained H9-derived NSCs for the dynein intermediate chain (DIC), the p150^*Glued*^ subunit of dynactin and NuMA during mitosis (Fig 3). All were present at the spindle poles and on the spindle, along astral microtubules and at cortical sites (Fig 3A–3C). We assessed the dependence of these distributions on HTT, by silencing the endogenous human HTT. When HTT was silenced, we did not observe obvious changes in total levels of p150^*Glued*^, DIC and NuMA proteins (S1C Fig). The silencing of HTT in WT-NSCs with the sh*HTT1*.*1* plasmid (Fig 1) resulted in the partial mislocalization of dynein and p150^*Glued*^ away from the spindle poles (Fig 3A–3C). By contrast, NuMA accumulated at the spindle poles when HTT levels were decreased. In HTT-depleted human NSCs, lower levels of dynein, p150^*Glued*^ and NuMA were detected on the astral microtubules and in the cell cortex. These data suggest that human HTT controls the correct distribution of several key mitotic proteins, potentially influencing spindle orientation in human WT-NSCs.

### Human adult-onset HD mutations of the *HTT* gene have a dominant negative effect on spindle orientation in HD-hESC-derived NSCs

**Fig 4:**
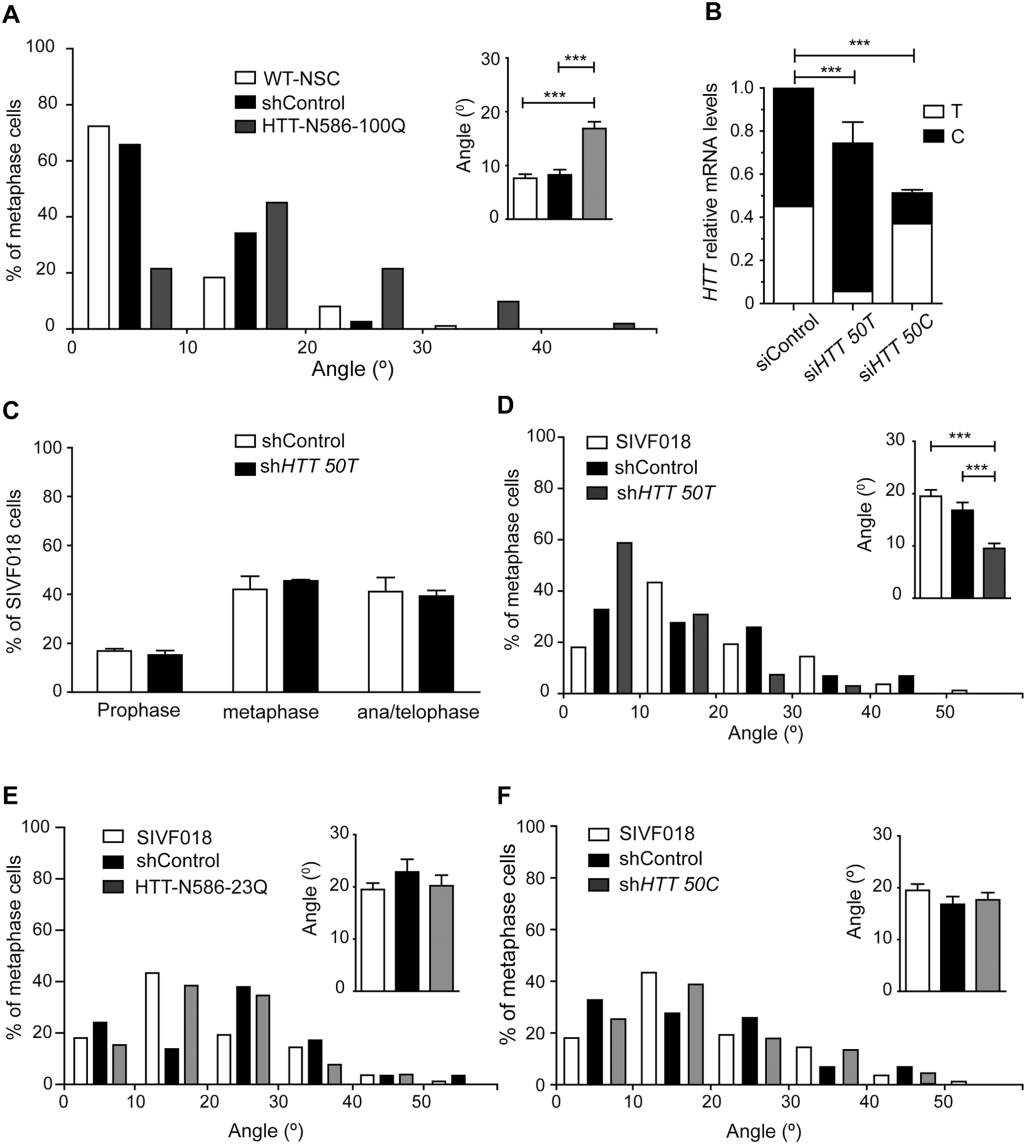
Allele-specific silencing rescues spindle misorientation in NSC derived from HD-hESC. (A) H9-hESC derived neural stem cells were either non-electroporated (WT-NSC) or electroporated as indicated. Distribution and average of spindle angles of metaphase cells are shown. WT-NSC: *n* = 86, WT-NSC shControl: *n* = 51, WT-NSC HTT-N586-100Q: *n* = 38 cells were analysed. (B) *HTT* allelic ratio measured by RT-qPCR in SIVF018-hESC derived HD-NSCs transfected as indicated every 48 hours; mRNAs were collected at day 7. (C) Distribution of shControl and sh*HTT50T*-treated SIVF018 HD-NSC in prophase, metaphase and ana/telophase. shControl: *n* = 629, sh*HTT*50T *n* = 402 cells were analysed. (D, E) SIVF018 HD-NSCs were either non-electroporated or electroporated as indicated. Distribution and average of spindle angles of metaphase cells are shown. (D): SIVF018: *n* = 83, shControl: *n* = 61, sh*HTT50T*: *n* = 68. (E) SIVF018: *n* = 83, shControl: *n* = 61, HTT N586-23Q: *n* = 29 cells were analysed. (F) SIVF018 HD-NSCs were non-electroporated or electroporated as indicated. Distribution and average of spindle angles of metaphase cells are shown. SIVF018: *n* = 83, shControl: *n* = 61, sh*HTT50C*: *n* = 67 cells were analysed. All data are from at least 3 independent experiments. Results are shown as mean values ± SEM. ***p<0.001; Student’s t-test.

We then investigated the effect of HD mutations on the role of HTT during spindle orientation in dividing NSCs derived from hESCs. We first expressed a mutant 586-amino acid N-terminal fragment of HTT with a large expanded polyQ stretch of 100 residues (N586-HTTQ100) in WT-NSCs derived from H9-hESCs. WT-NSCs expressing N586-HTT-Q100 had a significantly larger mean spindle angle than control cells (16.9° ± 1.3; *n* = 38 *versus* non-treated cells: 7.6° ± 0.7, *n* = 86 and WT-NSCs expressing an empty plasmid: 8.3#x00B0; ± 1, *n* = 51) (Fig 4A). In particular, 78.4% of cells in metaphase expressing mutHTT had a spindle angle exceeding 10°. We then used NSCs differentiated from a hESC line characterized as carrying one *HTT* allele with an adult-onset HD mutation during a pre-implantation genetic diagnosis procedure (SIVF018 line). In this cell line, the longest CAG expansion contained 46 CAGs. The size of the CAG expansion and the link between the mutated allele and the rs362331 SNP at exon 50 (50T) were confirmed (S1A Fig). The allelic ratio for *HTT* was determined with a *Taq*man probe specific for the rs362331-T and -C SNP in HD-NSCs nucleofected with siRNA targeting the rs362331-T (si*HTT50T*) and rs362331-C (si*HTT50C*) SNPs. This confirmed that SNP-targeting RNA interference could discriminate between the two alleles, as shown in a previous study [16]. Total HTT expression (i.e both alleles), as assessed by RT-qPCR, was reduced by 35.3% ± 1.4 (*n* = 4) in SIVF018-derived NSCs after transfection with the si*HTT50T* plasmid, and by 24.60% ± 2.8 (*n* = 4) in SIVF018-derived NSCs after transfection with the si*HTT50C* plasmid (Fig 4B). In agreement, total HTT and mutant HTT protein levels were decreased (S1D and S1E Fig).

The expression of a mutant HTT fragment leads to a defect in spindle orientation resembling the loss of function in WT-NSCs (Figs 2B and 4A). We therefore assessed whether the allele-specific silencing of mutant HTT was associated with a recovery of function. The treatment of SIVF018 HD-NSCs with sh*HTT50T* had no effect on progression through the cell cycle (Fig 4C). We then compared spindle angles in untreated, and shControl- or sh*HTT50T*-treated SIVF018 cells. Untreated and shControl-treated SIVF108 HD-NSCs had spindle angle distributions and mean spindle angles consistent with the loss of function (19° ± 1.2, *n* = 83; 16.8° ± 1.5, *n* = 61) (Fig 4D; see also Fig 2). By contrast, most sh*HTT50T*-treated SIVF018 cells divided with a spindle angle of less than 10°, and the mean spindle angle was similar to that in wild-type cells (9.5° ± 1, *n* = 68). We also expressed a wild-type HTT fragment (N586-HTT-Q23) in mutant cells, but this fragment had no effect on the distribution of spindle angles or on the mean spindle angle in the mutant cells (Fig 4E). In SIVF018 NSCs transfected with sh*HTT*50C, spindle angle was similar to that in controls (Fig 4F). These results indicate that a limited expansion of the N-terminal polyglutamine tract in HTT similar to that found in most patients with adult-onset HD is sufficient to induce a dominant-negative effect on spindle orientation. Furthermore, allele-specific silencing of mutant HTT in HD cells was found to be associated with a recovery of function during spindle orientation.

### Recovery of dynein, p150^*Glued*^ and NuMA localization after mutant HTT allele-specific silencing

**Fig 5:**
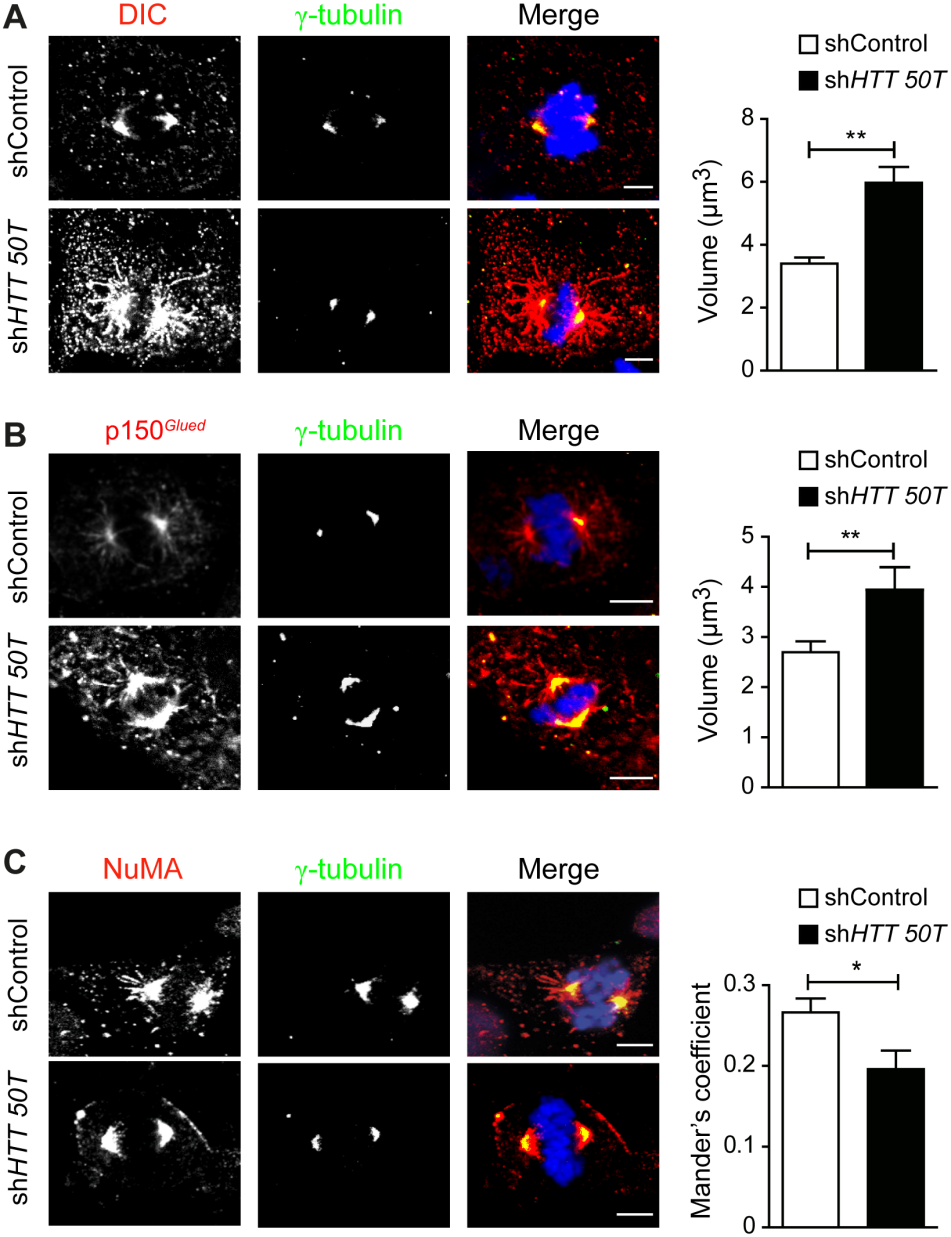
Allele-specific silencing of mutant HTT rescues dynein, p150^*Glued*^ and NuMA mislocalization in human HD-NSCs. Immunostaining of shControl and sh*HTT50T*-treated metaphasic neural stem cells with anti-DIC (A), anti-p150^*Glued*^ (B) or anti-NuMA (C) and anti-γ-tubulin antibodies. Nuclei are counterstained with DAPI. Scale bars, 5 μm. Graphs correspond to the quantification of (A): shControl: *n* = 46 and sh*HTT50T*: *n* = 46; (B): shControl: *n* = 54 and sh*HTT50T*: *n* = 45; (C): shControl: *n* = 16 and sh*HTT50T*: *n* = 28 cells analysed in at least 3 independent experiments. Results are shown as mean values ± SEM. ***p<0.001, **p<0.01, *p<0.05; Student’s t test.

We then investigated whether the functional rescue of spindle orientation induced by the sh*HTT50T* treatment of SIVF018 HD-NSC affected the localization of dynein, p150^*Glued*^ and NuMA. Cells were treated with shControl and sh*HTT50T* and immunolabeled for these proteins (Fig 5). In mutant cells treated with the control construct, the patterns of staining for dynein, p150^*Glued*^ and NuMA were approximately similar to those in wild-type neural cells in which HTT was silenced (compare Figs 3 and 5). In mutant cells treated with sh*HTT50T*, the localization of these proteins was restored to that observed in WT-NSCs. Indeed, there was more dynein and p150^*Glued*^ at spindle poles and along astral microtubules in SIVF018 HD-NSCs transfected with sh*HTT50T*, than in cells transfected with shControl (Fig 5A and 5B). Following the treatment of SIVF018 HD-NSCs with sh*HTT50T*, the spindle pole signal of NuMA was less dispersed than that in shControl-treated cells (Fig 5C).

Our results show that allele-specific silencing selectively rescues the displacement of dynein, dynactin and NuMA. Our data suggest that allele-specific silencing rescues the phenotype of HD cells by restoring HTT function.

## Discussion

We show here that HTT interferes with the spindle orientation of human neural stem cells and is required for the correct mitotic distribution of several key components of the spindle. We tested the hypothesis that the heterozygous adult-onset mutations responsible for the vast majority of cases of HD in patients result in a dominant-negative effect of the mutant-HTT on the function of wtHTT in correct spindle orientation and dynein, p150^*Glued*^ and NuMA localization to the spindle pole and cell cortex in human mutant neural cells.

Previous studies have established a role for HTT in the regulation of molecular motors. HTT acts as a scaffold protein for the dynein/dynactin complex in various types of dividing and post-mitotic cells in *Drosophila* and mouse. This complex of HTT and microtubule-associated proteins is essential for microtubule-dependent vesicular transport in axons [9], [10], spindle assembly and maintenance [12], and apical trafficking of the PAR-polarity complex in mammary epithelium [29]. We show here that human HTT regulates spindle orientation in dividing neural cells and is required for the correct localization of dynein, p150^*Glued*^ and NuMA.

We previously showed that mutHTT impairs mitotic spindle orientation and alters the mitotic distribution of key components of the spindle. However, this interference was demonstrated in mouse embryos or embryo-derived cells carrying two mouse mut*HTT* alleles with 111 CAGs (*Hdh*^*Q111/Q111*^ knock-in mouse model) or in human cancer cells (Hela) overexpressing an N-terminal fragment of human HTT with 68 CAGs [22]. The *Hdh*^*Q111/Q111*^ knock-in mouse model displays temporally and spatially appropriate levels of *mutHTT* expression, but both these models resemble rare and exacerbated forms of juvenile-HD more closely than the most common (>90% patient) adult-onset form of HD. We found that a heterozygous adult-onset HD mutation of the *HTT* gene with only 46 CAGs was also able to interfere with mitotic spindle orientation and the mitotic distribution of key components of the spindle in human neural cells. These results are consistent with our previous report on mutant HTT interference with the anterograde and retrograde axonal transport of BDNF vesicles in human neurons carrying adult-onset HD mutations [16].

The importance of asymmetric cell divisions for stem cells/progenitors has been established in several tissues [30], [31]. In vertebrates, a correlation between spindle orientation and the acquisition of cell fate has been proposed for the division of skin progenitors [32] and neuronal radial glial cells (reviewed in [30], [31]). Consistent with the effect of wtHTT on spindle orientation, juvenile-mutant HTT in *Hdh*^*Q111/Q111*^ knock-in mouse affects cortical progenitor cell division and neocortex development in mice [22]. These findings provide support for the notion that development, particularly that of the brain, may be abnormal in HD carriers. They are also consistent with the smaller adult intracranial brain volume measured in non-symptomatic carriers of adult-onset HD mutations and the smaller head size of children at risk of adult-onset HD [33], [34]. The demonstration that HD-iPSCs derived from somatic cells of patients with juvenile-HD (109–180 CAGs) had significantly more nestin-expressing cells (i.e immature progenitors) than controls after extensive neuronal differentiation *in vitro* is also consistent with the occurrence of subtle neurodevelopmental defects in HD [18].

Carroll and coworkers (2011) developed antisense oligonucleotides (ASO) targeting both exonic and intronic SNP sites that were able to decrease mutHTT levels in primary human cells, cultured primary neurons and the adult brain of YAC18 and BACHD mice [35]. Similarly, we showed that the selective degradation of *mutHTT* transcripts, with the preservation of *wtHTT* transcripts, could be achieved by targeting heterozygous exonic SNPs by plasmid- or lentivirus-based transfection with a specific shRNA [16]. In HD-hESC-derivatives, used as a cellular model of HD, we have shown that neurons derived from such hESC lines carrying adult-onset HD mutations reproduce the defect in anterograde and retrograde BDNF vesicular transport associated with HD [9]. This specific impairment of molecular motor function was rescued only by silencing of the *mutHTT* allele, with silencing of the *wtHTT* allele having no effect on the mean velocity of BDNF vesicles [16]. In the study reported here, we explored the spindle orientation dysfunction in dividing human neural cells mediated by the interference of mutHTT with molecular motor functions. By combining more systematic gain-of-function, pan-allelic and allele-specific loss-of-function approaches in human cells with wild-type and adult-onset HD alleles of *HTT*, we showed that the mutHTT isoform exerted a dominant-negative effect on the role of the wtHTT protein (encoded by the wild-type allele) in molecular motor function. The selective inactivation of *mutHTT* rescued spindle abnormalities in HD-NSCs.

In conclusion, our data confirm the relevance of neural derivatives of human pluripotent stem cells with HD mutations for the modeling of early HD phenotypes and for explorations of the potential of gene or drug-based therapies. We also show that the systematic use of extreme juvenile-onset HD mutations (as opposed to the most common HD mutations giving rise to 40–50 CAGs) is both unnecessary and potentially suboptimal for modeling of the cellular dysfunctions likely to contribute to the pathological features of HD in most patients. Finally, our data, together with previous reports of the selective (allele-specific) silencing of the mutHTT isoform, highlight the dominant-negative activity of mutHTT and suggest that gene therapy approaches based on the pan-allelic silencing of *HTT* may have limited therapeutic potential because they deal only with the adverse effects due to the directly toxic activities of mutHTT, but not the dominant-negative effects of mutHTT on wtHTT.

## Supporting Information

S1 Fig CAG repeat size and HTT expression levels in different experimental conditions. (A) CAG repeats size detection for H9 and SIVF018 obtained using a microfluidic chip from Agilent^®^ and respective sequencing showing the SNP rs362331 at exon 50. (B) Immunoblotting with anti-HTT (D7F7) and anti-α-tubulin antibodies of lysates of WT-NSC cells and WT-NSC cells treated with sh*HTT50T*. The graph represents the quantitative assessments of the ratio of HTT over tubulin in each condition (3 independent experiments; *p<0.01). (C) Immunoblotting with anti-HTT, NuMA, p150^*Glued*,^ anti-DIC and anti-α-tubulin antibodies of lysates of WT-NSC cells treated as indicated. The graph represents the quantitative assessments of the ratio of the indicated proteins over tubulin in each condition (3 independent experiments; ** p<0.01). (D) Immunoblotting with anti-HTT (D7F7) and anti-α-tubulin antibodies of lysates of SIVF018 cells and SIVF018 cells treated with sh*HTT50T*. (E) Immunoblotting with anti-HTT (1C2; recognizing the 46Q polyglutamine strech) and anti-α-tubulin antibodies of lysates of WT-NSC, SIVF018 and SIVF018 cells treated with sh*HTT50T* (50T).
(TIF)

S1 Table Sequences of the oligonucleotides used to generate the shRNA targeting the SNP and the control. The sense and anti-sense strands of the shRNA are shown in bold and the position of the SNP in red [36].
(DOCX)
