# Supplementary figures and images for: Dominant-Negative Effects of Adult-Onset Huntingtin Mutations Alter the Division of Human Embryonic Stem Cells-Derived Neural Cells
Source: PLoS One. 2016 Feb 10;11(2):e0148680. doi: 10.1371/journal.pone.0148680 (PMC4749329; doi:10.1371/journal.pone.0148680)

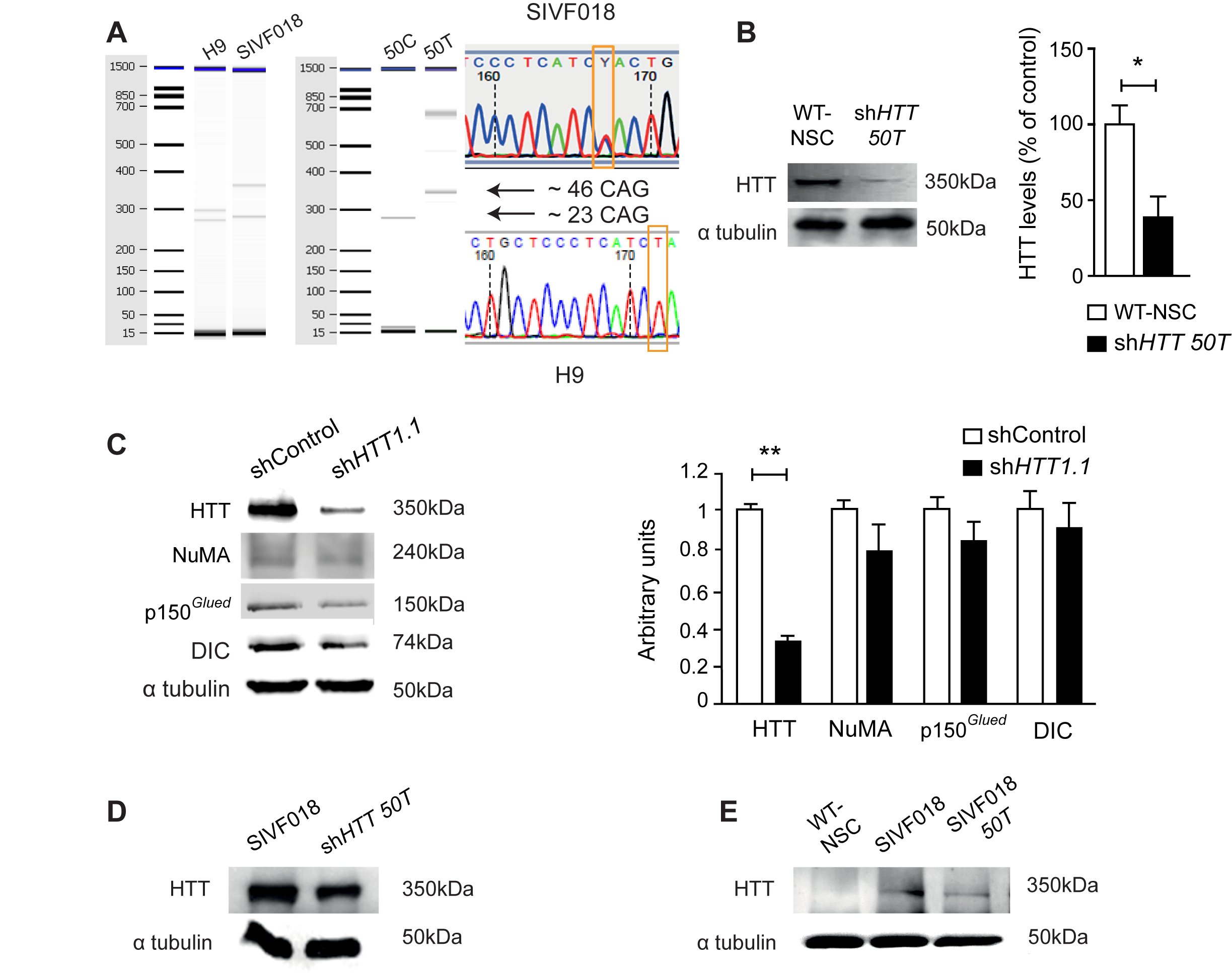

Supplement: S1 Fig — (A) CAG repeats size detection for H9 and SIVF018 obtained using a microfluidic chip from Agilent® and respective sequencing showing the SNP rs362331 at exon 50. (B) Immunoblotting with anti-HTT (D7F7) and anti-α-tubulin antibodies of lysates of WT-NSC cells and WT-NSC cells treated with shHTT50T. The graph represents the quantitative assessments of the ratio of HTT over tubulin in each condition (3 independent experiments; *p<0.01). (C) Immunoblotting with anti-HTT, NuMA, p150Glued, anti-DIC and anti-α-tubulin antibodies of lysates of WT-NSC cells treated as indicated. The graph represents the quantitative assessments of the ratio of the indicated proteins over tubulin in each condition (3 independent experiments; ** p<0.01). (D) Immunoblotting with anti-HTT (D7F7) and anti-α-tubulin antibodies of lysates of SIVF018 cells and SIVF018 cells treated with shHTT50T. (E) Immunoblotting with anti-HTT (1C2; recognizing the 46Q polyglutamine strech) and anti-α-tubulin antibodies of lysates of WT-NSC, SIVF018 and SIVF018 cells treated with shHTT50T (50T). (TIF) [file pone.0148680.s001.tif]
