# Supplementary material for: Dominant-Negative Effects of Adult-Onset Huntingtin Mutations Alter the Division of Human Embryonic Stem Cells-Derived Neural Cells
Source: PLoS One. 2016 Feb 10;11(2):e0148680. doi: 10.1371/journal.pone.0148680 (PMC4749329; doi:10.1371/journal.pone.0148680)
Supplement: S1 Table — The sense and anti-sense strands of the shRNA are shown in bold and the position of the SNP in red [36]. (DOCX) [file pone.0148680.s002.docx]

| **NAME** | **SEQUENCE** |
| --- | --- |
| **sh*HTT1.1.*** | CTAGTTTCCAAAAA**AAGAACTTTCAGCTACCAA**TCTCTTGAA**TTGGTAGCTGAAAGTTCTT**GGGGATCTGTGGTCTCATACAGAAC |
| **shControl** | CTAGTTTCCAAAAA**GTATCGATCACGAGACTAG**TGACAGGAAG**CTAGTCTCGTGATCGATAC**GGGGATCTGTGGTCTCATACAGAAC |
| **si/**  **sh*HTT50C*** | CTAGTTTCCAAAAA**CCCTCATCCACTGTGTGCA**TGACAGGAAG**TGCACACAGTGGATGAGGG**GGGGATCTGTGGTCTCATACAGAAC |
| **si/**  **sh*HTT50T*** | CTAGTTTCCAAAAA**CCCTCATCTACTGTGTGCA**TGACAGGAAG**TGCACACAGTAGATGAGGG**GGGGATCTGTGGTCTCATACAGAAC |
